# Visits to Private Asylums

**Published:** 1903-12-12

**Authors:** 


### VISITS TO PRIVATE ASYLUMS.

By Our Rambling Reporter.

### KINGSDOWN HOUSE, BOX, NEAR BATH.

This private asylum is situated in Wiltshire, about fifteen minutes' walk from the Box station on the Great Western line; but it may also be reached by a five-mile drive from Bath. The house is placed on the brow of a hill about four hundred feet above the sea-level, and from it there are splendid views of the surrounding country. This delightful site gives the whole institution an air of freedom; and this feeling is accentuated on entering the house, where it is found that everything has been done to preserve a home-like appearance, and to obtain for the patients as much freedom as is at all consistent with their being carefully and properly looked after. The mansion itself is no parvenu. It was at one time the residence of Judge Jeffries of "hanging" notoriety, and an old oil-painting of that celebrity still occupies a prominent place in the hall, where, too, are seen numerous other works of art.

Insane patients were first admitted here in 1798, so that it must be one of the oldest licensed houses in England. But what a change has taken place in its surroundings since those early days when the medical treatment of insanity was of the crudest possible description, and the modern humane treatment was just beginning to dawn in France! Six years earlier Pinel had entered the Bicêtre; but it was not until thirty-seven years after the opening of Kingsdown House as an asylum that Gardiner Hill inaugurated the non-restraint system at Lincoln. It is said that walls have ears. It is a pity they have not pens at times, so that they could have left us a veritable account of what passed within our asylums previous to the Victorian age. Naturally, many improvements would be gradually carried out at Kingsdown House; but it was reserved for the present proprietor to complete them and to bring the whole institution into harmony with modern ideas as a private residence for the insane. The water-supply has been placed on a very satisfactory basis; appliances for the prevention of the spread of fire and for the safety of the patients are all that can be desired; lighting by gas has been substituted for the old plan of oil lamps; hot-water radiators have been fitted up; the airing court and boundary walls have been pulled down and light iron fencing substituted in places where any fence at all was needed. The old courts are now turned into pretty gardens and lawns, but very few of the patients are confined to the grounds for their exercise and recreations, as they are allowed to walk along the roads and lanes. In carrying out the various improvements inside and outside the house Dr. MacBryan has expended upwards of £5,000.

So far as we are aware, this is the only private asylum which issues an annual report after the manner of our county asylums. From it we learn that 24 patients were admitted last year; that 29 were discharged, and that 3 died. The average daily number resident was 35. Of the cases admitted 10 suffered from melancholia; 2 from acute mania; 6 from mania; 4 from delusional insanity, and 1 from general paralysis of the insane. The recoveries stand at the high percentage of 54·1 of the admissions. Restraint is never used at Kingsdown House, and seclusion but rarely. There are nice old-fashioned drawing-rooms, dining-rooms, and a billiard-room; and many of the single-bedded rooms are of large size and are well furnished. There are plenty of books for the patients to read. Dr. MacBryan, who had long experience in one of our county asylums, rightly discourages idleness among his patients; and he tells us that 42 per cent. of the ladies are employed. Among the men the percentage is much lower; but that is owing to the serious forms of brain diseases from which at the present time the majority of the men are suffering, and which precludes the idea of their employment.

The house is licensed for 33 ladies and 10 men. The fees range from £2 2s. to £5 5s. a week, according to the nature of the case and the kind of accommodation required.

The Rev. W. White, Vicar of Box, officiates as chaplain, and holds a service once a week in the asylum. Many of the patients attend the parish church on Sundays.

As above stated, the asylum is licensed for 43 patients; and for this number a complete staff of higher officials must be kept up. Kingsdown House is therefore another of our private asylums which should be allowed to double the number it is at present permitted to receive. The existing building is of such a plan that it could easily have wings for forty or fifty patients added to it. A much larger number of patients paying low rates could then be received; and it is accommodation at £2 2s., or even less, that is so urgently called for in some districts. Bath itself has 50,000 inhabitants; and it is within two hours' run of London.

